# Supplementary material for: Adipocyte Expression of O-Glycoprotein Procollagen C-Endopeptidase Enhancer Protein 2 (PCPE2): Mechanisms Linking Fibrosis and Beiging of White Adipose Tissue
Source: bioRxiv. 2026 Jul 26:2026.07.24.740318. Preprint. [Version 1] doi: 10.64898/2026.07.24.740318 (PMC13419799; doi:10.64898/2026.07.24.740318)
Supplement: 1 [file NIHPP2026.07.24.740318V1-supplement-1.pdf]

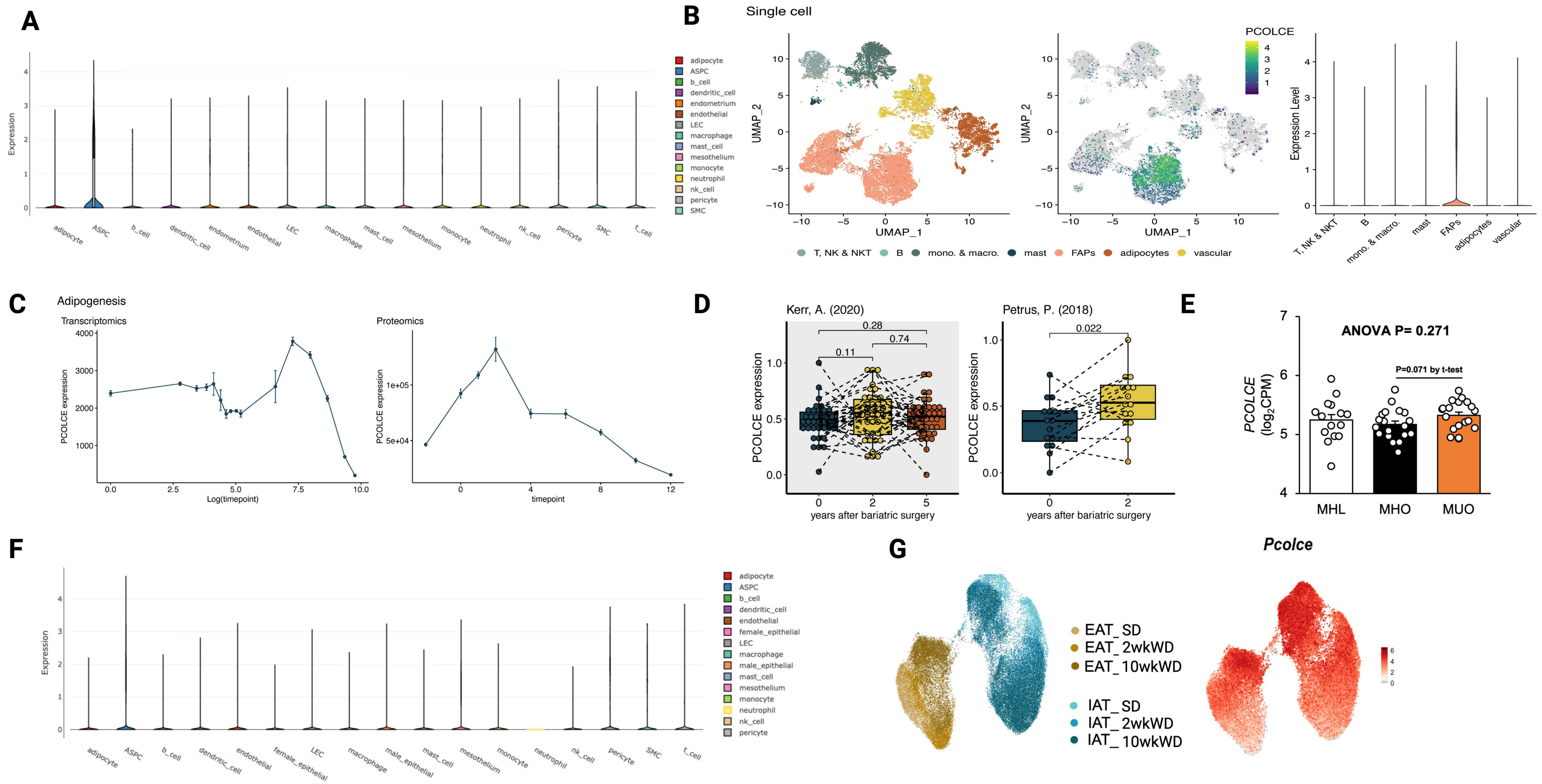

Supplementary Figure 1. Expression of *PCOLCE* / *Pcolce* in Adipose Tissue in Humans and Mice. **(A)** *PCOLCE* relative expression among various cell types as determined by single-cell RNA sequencing (scRNA-seq) of human white adipose tissue (WAT) **(B)** UMAP of human WAT *PCOLCE* expression in fibroinflammatory adipocyte precursors (FAPs) and mature adipocytes relative to various immune cells **(C) (Left panel)** *PCOLCE* mRNA expression and **(Right panel)** PCPE protein expression expressed during adipocyte differentiation **(D)** Human *PCOLCE* mRNA expression from subcutaneous adipose tissue (SAT) collected before and after weight loss. Studies involved obese individuals who underwent bariatric surgery, with follow-up assessments conducted **(Left panel)** two and five years post-surgery as reported by Kerr et al., 2020 or **(Right panel)** two years post-surgery in the study reported by Petrus et al., 2018 **(E)** *PCOLCE* mRNA levels from human patients designated metabolically healthy lean (MHL; n=19), metabolically healthy obese (MHO; n=18) and metabolically unhealthy obese (MUO; n=14). Data are presented as mean ± SEM. Statistical significance was determined by one-way ANOVA **(F)** scRNA-seq of mouse WAT showing *Pcolce* expression among various cell types **(G) (Left Panel)** UMAP from public scRNA-seq dataset (Nahmgoong, Hahn, et al., 2022) showing mouse adipose stem cells from epididymal adipose tissue (EAT) and inguinal adipose tissue (IAT) from three dietary conditions: normal standard diet (SD), 2-week Western diet (2wWD), and 10-week Western diet (10wWD). Colors indicate tissue origin and dietary condition. **(Right panel)** UMAP of *Pcolce2* gene expression with dietary conditions. **Note:** *PCOLCE* / *Pcolce* scRNA-seq data of human **(A)** and mouse **(G)** were obtained from the single cell portal (Broad Institute of MIT and Harvard; Tarhak et al., 2025), based on datasets from Emont et al., 2022. Adipogenesis data and UMAP of human scRNA-seq **(B and C)** were acquired from from the adipose tissue Knowledge portal (Zhong et al., 2025), which includes data from Massier et al., 2023; Hinte et al., 2024; and Reinisch et al., 2024.

bioRxiv preprint doi: https://doi.org/10.64898/2026.07.24.740318; this version posted July 26, 2026. The copyright holder has placed this preprint (which was not certified by peer review) in the Public Domain. It is no longer restricted by copyright. Anyone can legally share, reuse, remix, or adapt this material for any purpose without crediting the original authors.

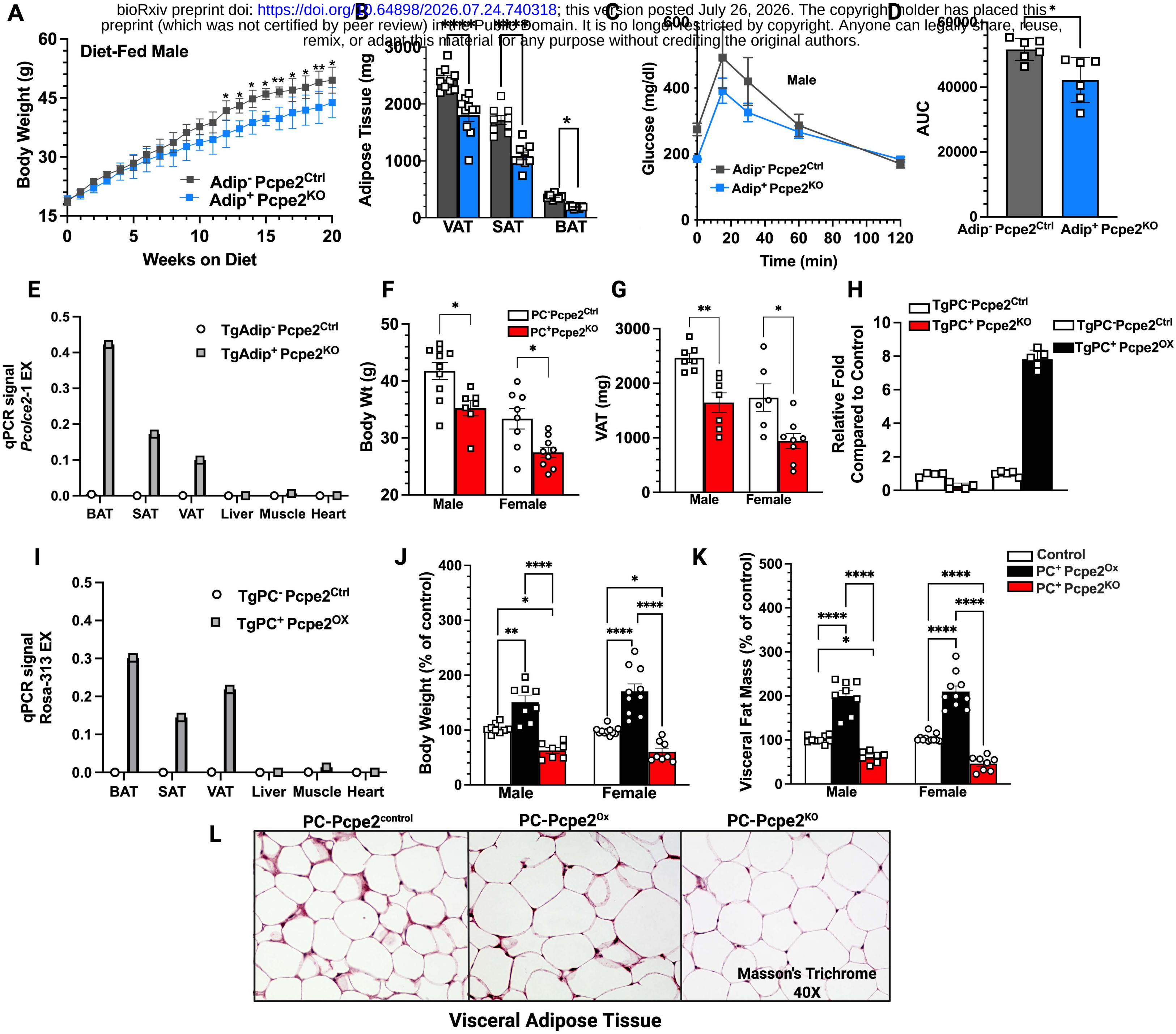

Supplementary Figure 2. Pcpe2 Expression and Specificity of Cre Recombinase. Adip^{+}Pcpe2^{KO} and Adip^{-}Pcpe2^{Ctrl,} control mice were fed a WD for 20 weeks. **(A)** Body weight of WD male mice from indicated genotype **(B)** End point body weight in male mice **(C)** Glucose tolerance test (GTT) in WD male mice as compared to their controls. **(D)** Area under the curve (AUC) for GT **(E)** Indicated mouse tissues were submitted to Transnetyx Genotyping Services for DNA analysis using specific probes developed for each mouse strain. The qPCR signal for the probe detecting *Pcolce2* floxed exon 3, *Pcolce2*-1 EX, targets gene sequence after Cre recombinase removes exon 3 of the *Pcolce2* gene, thus, a positive signal indicates removal of the region indicated the specificity of the adiponectin Cre recombinase. Similar tissue specificity were noted when *Pcolce2* floxed mice were crossed with the Pdgfra (PC) Cre (data not shown) **(F-G)** Body and VAT weights in 20- weeks WD-fed male and female mice comparing TgPC^{+}Pcpe2^{KO} and their negative littermate controls. Showing similar weight changes regardless of Cre recombinase **(H)** VAT Pcpe2 mRNA abundance from male mice fed SD from indicated genotype, shows Pcpe2 overexpression in TgPC^{+}Pcpe2^{OX} mouse stain compared to its control **(I)** Indicated mouse tissues were submitted to Transnetyx Genotyping Services for DNA analysis using specific probes developed for each mouse strain. Transgenic mice expressing Pdgfra (PC^{+}) were crossed with *Pcolce2* overexpressor mice, TgPC $^{+}$Pcpe2^{OX} mice, and their negative PC^{-} controls PC^{-}Pcpe2^{Ctrl}. For this strain the PCR signal is plotted for the probe detecting Rosa-313 EX, which targets the floxed DNA sequence corresponding to the stop codon which is removed after Cre recombinase allowing overexpression of the *Pcolce2* gene. Thus, a positive signal indicates that the stop codon has been removed **(J-K)** Body and VAT weights expressed as percent of control (PC^{-} littermate control used for each mouse strain) from 20-25 week WD fed male and female mice of the indicated genotype. **(L)** Representative images of VAT sections stained with Masson's Trichrome from indicated genotype. All data represent the mean ± SEM (n=5-15). Statistical significance were analyzed using unpaired t-test or one-way ANOVA followed by Tukey's post hoc test. *$p \leq 0.05$.

bioRxiv preprint doi: https://doi.org/10.64898/2026.07.24.740318; this version posted July 26, 2026. The copyright holder has placed this preprint (which was not certified by peer review) in the Public Domain. It is no longer restricted by copyright. Anyone can legally share, reuse, remix, or adapt this material for any purpose without crediting the original authors.

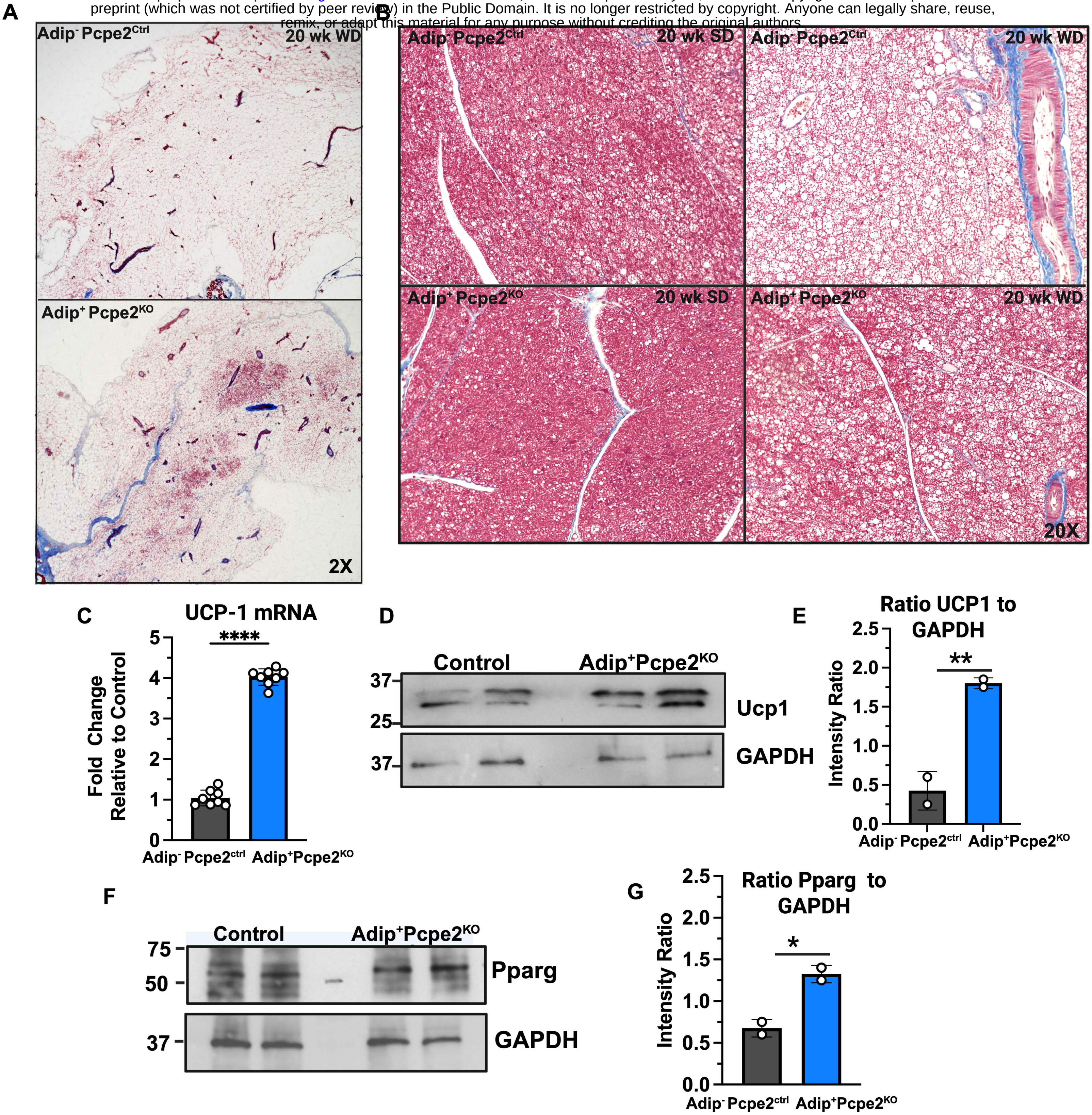

Supplemental Figure 3. Absence of Pcpe2 Promotes WAT Beiging and Attenuates BAT Whitening. **(A)** Representative images of Masson's Trichrome stained WAT 2X images from Adip$^-$Pcpe2^{Ctrl} **(top panel)** and Adip$^+$Pcpe2^{KO} **(bottom panel)** mice fed WD for 20 weeks **(B)** Representative Masson's Trichrome stained BAT images from Adip$^-$Pcpe2^{Ctrl} (**top panels)** and Adip$^+$Pcpe2^{KO} **(bottom panels)** mice fed SD **(left panels)** or WD **(right panels)** for 20 weeks **(C)** UCP1 mRNA abundance in SAT of indicated mouse genotype **(D)** Western blot analysis of Ucp1 and glyceraldehyde phosphate dehydrogenase (GAPDH) from SAT of WD-fed mice of indicated genotype **(E)** Quantification of band intensity as a rato of UCP1 to GAPDH **(F)** Western blot analysis of Pparg and GAPDH from SAT of WD-fed mice of indicated genotype **(G)** Quantification of band intensity as a ratio of Pparg to GAPDH. PCR data represent mean ± SEM (n=8) Statistical significance was determined by unpaired t-test. Western blot lanes show protein extracts from two different animals of each genotype.

bioRxiv preprint doi: https://doi.org/10.64898/2026.07.24.740318; this version posted July 26, 2026. The copyright holder has placed this preprint (which was not certified by peer review) in the Public Domain. It is no longer restricted by copyright. Anyone can legally share, reuse, remix, or adapt this material for any purpose without crediting the original authors.

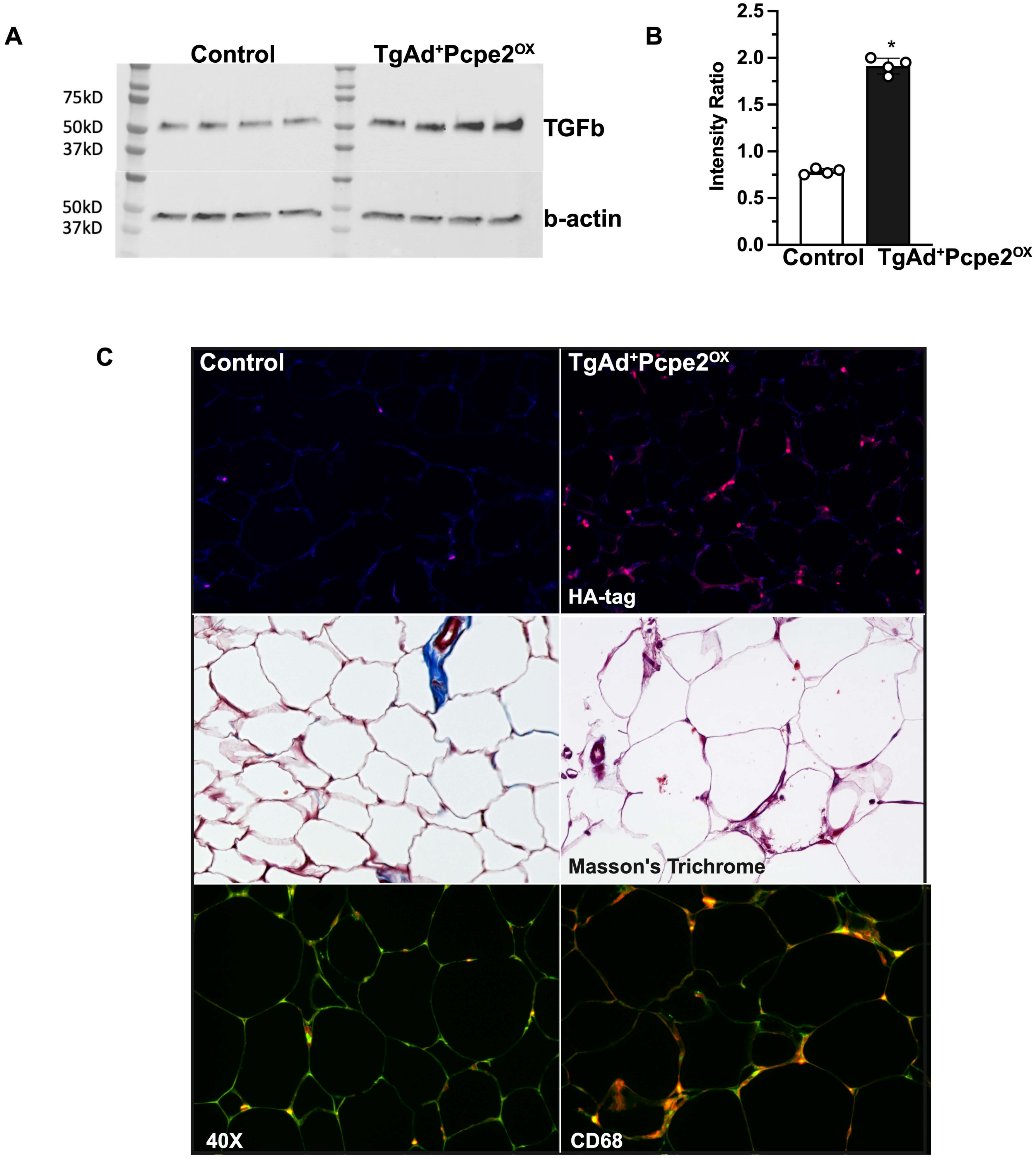

Supplemental Figure 4. Pcpe2 Overexpression Enhances TGFb Signaling and Inflammation in VAT. FAPs were isolated from VAT SVF using collagenase digestion and then isolated by FACS sorting and cultured for 4 days. Cell were treated with 2 ng/ml of TGFb1 for 90 min after which the culture medium was collected and subjected to Western analysis with intensity ratio quantification **(A)** Sections of re-harvested transplanted VAT were stained for hemagglutinin antigen (HA) to detect the fusion protein Pcpe2-HA tag expressed in TgAd+Pcpe2^{Ox} mice (**top panels**), Masson's trichrome (**middle panels**), CD68 and perilipin **(bottom panels).**

bioRxiv preprint doi: https://doi.org/10.64898/2026.07.24.740318; this version posted July 26, 2026. The copyright holder has placed this preprint (which was not certified by peer review) in the Public Domain. It is no longer restricted by copyright. Anyone can legally share, reuse, remix, or adapt this material for any purpose without crediting the original authors.

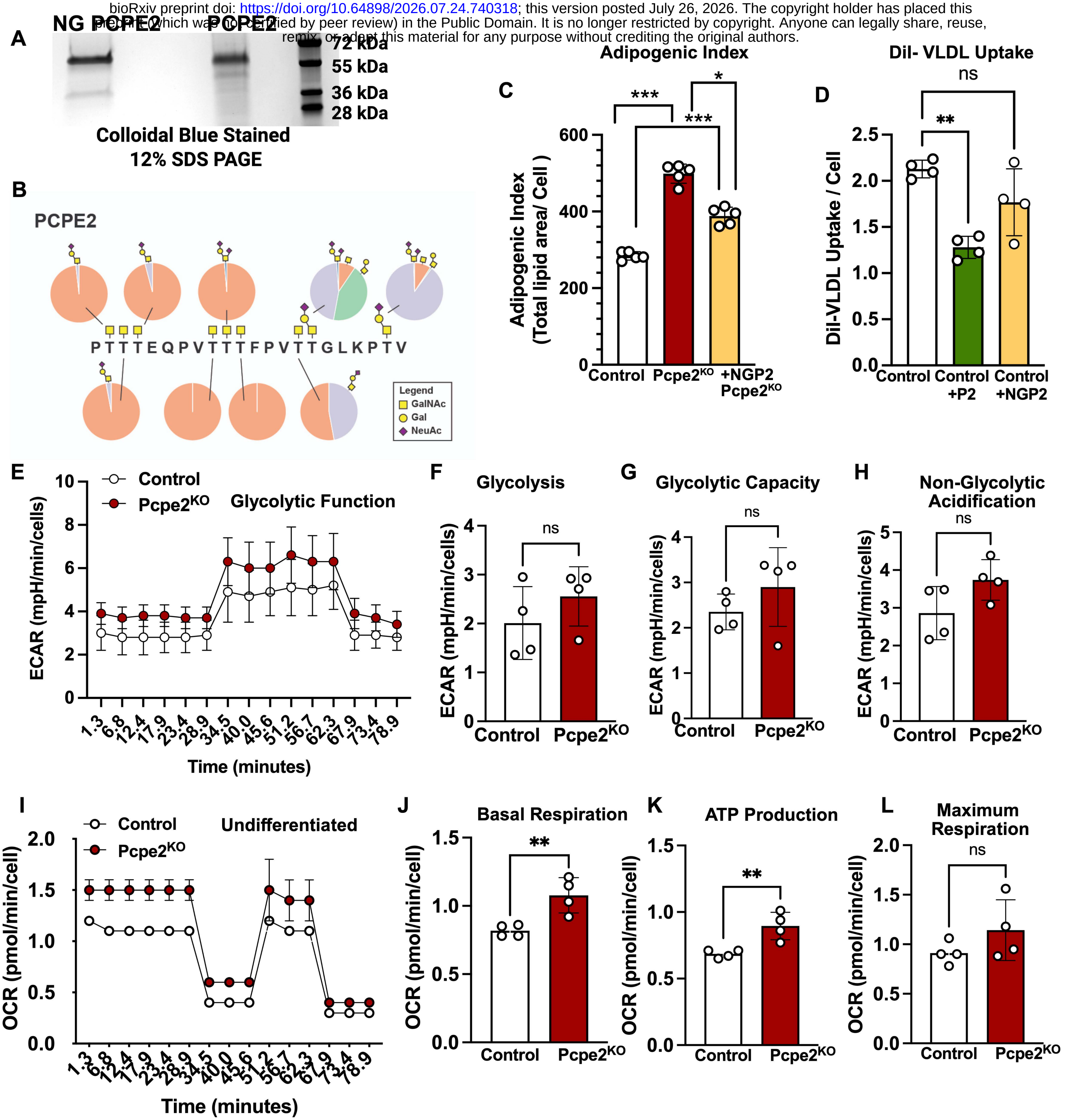

Supplemental Figure 5. PCPE2 Glycosylation and Adipogenic Index (A) PCPE2 and NG PCPE2 were purified from ExpiCHO expression system and aliquots applied to 12% SDS PAGE, then stained with colloidal blue **(B)** Mass spectrometry analysis showing glycans associated with indicated amino acids within linker region. Pie chart shows relative abundance for each O-glycan detected **(C)** Adipogenic index from *ex vivo* differentiated adipocytes from control or Pcpe2^{KO} mouse SAT SVF **(D)** DiI-VLDL uptake in differentiated adipocytes from indicated genotype normalized to total cell number per well **(E)** Seahorse study showing extracellular acidification rate traces from differentiated adipocytes from control and Pcpe2^{KO} SAT SVF **(F)** Glycolysis **(G)** Glycolytic capacity **(H)** Non-glycolytic acidification **(I)** Seahorse study showing oxygen consumptKon rate curve from undifferentiated SVF cells derived from control and Pcpe2^{KO} SAT SVF **(J)** Basal Respiration **(K)** ATP Production **(L)** Maximum Respiration. All data represent mean ± SEM of differentiated adipocytes from SAT SVF with n=6 wells per genotype -96 well plate. Statistical significance was determined by unpaired t-test or ANOVA.
